# Induction of acute lung inflammation in mice with hemorrhagic shock and resuscitation: role of HMGB1

**DOI:** 10.1186/s12950-014-0030-7

**Published:** 2014-10-08

**Authors:** Raymond LC Kao, Xuemei Xu, Anargyros Xenocostas, Neil Parry, Tina Mele, Claudio M Martin, Tao Rui

**Affiliations:** Department of National Defense, Canadian Forces Health Services, Ottawa, ON Canada; Critical Care Western, Department of Medicine, Schulich School of Medicine and Dentistry, Western University, London, ON Canada; Division of Hematology, Department of Medicine, Schulich School of Medicine and Dentistry, Western University, London, ON Canada; Center for Critical Illness Research, Lawson Health Research Institute, 800 Commissioner’s Rd E, N6A 5 W9 London, ON Canada

**Keywords:** Hemorrhagic shock, Gut injury, Acute lung inflammation, HMGB1

## Abstract

**Background:**

Hemorrhagic shock and resuscitation (HS/R) can induce multiple organ failure which is associated with high mortality. The lung is an organ commonly affected by the HS/R. Acute lung injury is a major cause of dysfunction in other organ systems. The objective of this study is to test the hypothesis that HS/R causes increased gut permeability which results in induction of high mobility group box1 protein (HMGB1) and further leads to the development of acute lung inflammation.

**Materials and methods:**

A mouse model of HS/R was employed in this study. Gut permeability and bacterial translocation were assessed with circulating FD4 and lipopolysaccharide (LPS). Circulating HMGB1 was determined with ELISA. Acute lung inflammation (ALI) was determined with lung myeloperoxidase (MPO) activity and pulmonary protein leakage.

**Results:**

HS/R induced intestinal barrier dysfunction as evidenced by increased circulating FD4 and LPS at 30 min and 2 hrs after resuscitation, respectively. In addition, circulating HMGB1 levels were increased in mice with HS/R as compared with sham animals (p < 0.05). HS/R resulted in ALI (increased lung MPO activity and pulmonary protein leakage in mice with HS/R compared with sham mice, p < 0.05). Inhibition of HMGB1 (A-box and TLR4^−/−^) attenuated the ALI in mice with HS/R. However, inhibition of HMGB1 did not show protective effect on gut injury in early phase of HS/R in mice.

**Conclusions:**

Our results suggest that induction of HMGB1 is important in hemorrhagic shock and resuscitation-induced acute lung inflammation.

## Introduction

Traumatic injury is the leading cause of death in people who are under 45 years in the United States. Hemorrhagic shock remains to be the leading cause of mortality, accounting for 30-40% of trauma related death. Patients recovering from hemorrhagic shock frequently develop a systemic inflammatory response which can put the patients at risk for multiple organ failure (MOF) [1-3]. However, the exact mechanism(s) leading to MOF after the onset of hemorrhagic shock and resuscitation (HS/R) needs to be elucidated.

The gut is one of the most vulnerable organ to be injured during HS/R [4]. The gut mucosa is affected in HS/R, resulting in significant local inflammation and enterocyte injury, breech of the intestinal barrier and translocation of harmful mediators and pathogens from lumen into intestinal tissue. Gut ischemia during the hemorrhagic shock is believed to further induce remote organ dysfunction. Therefore, the injured gut serves to propagate systemic injury during HS/R and initiates the process for induction of MOF [4,5].

As previous studies indicate that HS/R induces systemic ischemia/reperfusion which causes injuries to affected tissues due to robustly generation of reactive oxygen species (ROS). The ROS leads to exaggerated systemic inflammatory responses and cytokine production. According to literature, neutrophils are important contributing factors to ROS and cytokine production. Thus, the accumulation of the activated neutrophils within the lungs has been reported as an important factor in induction of lung injury, and further progress to acute respiratory distress syndrome (ARDS) in the clinical setting [6,7].

High mobility group box1 protein (HMGB1) is an alarmin and cytokine which can be released from activated immune cells as well as stressed and/or necrotic cells, including cardiac myocytes, in response to tissue injury [8-10]. In general, HMGB1 exerts its biological roles through interaction with several HMGB1 receptors including: receptor for advanced glycation end products (RAGE), toll-like receptor-2 (TLR2), TLR4 and TLR9 [10]. However, HMGB1 needs to interact with TLR4 in order to induce a proinflammatory response [10]. We have demonstrated that HMGB1 is an important mediator in sepsis-induced myocardial dysfunction [8]; an increase in HMGB1 expression by cardiomyocytes after ischemia/reperfusion contributes to myocardial apoptosis [11]. However, the role of HMGB1 in HS/R-induced MOF, specifically, the development of lung inflammation after the HS/R is not clear.

By using a mouse model of HS/R, we tested the hypothesis that HS/R induces gut injury and further leads to increase in circulating levels of HMGB1; the latter effect results in lung inflammation and injury. The study is the first to provide mechanistic information which links HS/R-induced gut injury and subsequent acute lung inflammation/injury.

## Materials and methods

### Mice

C57BL/6 mice were obtained from Charles River Canada (St. Constant, PQ, Canada). TLR4^−/−^ mice on a C57BL/6 background were obtained from Jackson Laboratories. The mice were housed in Vivarium Service at Victoria Research Laboratories with a 12-hour light/dark cycle and free access to rodent chow and tap water. The experimental protocol followed the institution’s guide for the care and use of laboratory animals and was approved by the Western University Animal Care and Use Committee (Protocol No. 2011–028).

### Mouse model of hemorrhagic shock and resuscitation

Ten-week old, male mice were anesthetized with intraperitoneally (i.p.) injection of ketamine (120 mg/kg) and xylazine (4 mg/kg). Both the right jugular vein and the right carotid artery were cannulated. The jugular vein cannulation was used for the administration of heparin and resuscitation. The carotid artery cannulation was used for blood pressure monitoring and blood withdrawal. Hemorrhagic shock was initiated by blood withdrawal and a reduction of the mean arterial blood pressure (MAP) to 30 mmHg in 15 min. The blood was harvested into a 1-ml syringe with heparin to prevent coagulation. The MAP was kept at 30 mmHg for another 60 minutes. Subsequently, the mice were resuscitated with transfusion of 1.5 volume of Ringer’s Lactate (RL) over 10 min followed by transfusion of red blood cells derived from the shed blood diluted with 1 volume of RL. Subsequently, the catheters were removed, the blood vessels were ligated and incision closed [12]. In some experiments, mice were given A-box (300 μg/mouse) [8], a HMGB1 inhibitor, at the beginning of the shed blood transfusion. The sham mice underwent same surgical procedures without blood withdrawal and resuscitation.

### Circulating Lipopolysaccharide (LPS) and HMGB1

Mouse blood samples were collected into heparinized tubes. Blood plasma was obtained after centrifugation (4°C, 500 *g* for 5 min). The circulating levels of LPS and HMGB1 were determined using a chromogenic Limulus Amebocyte Lysate (LAL) endotoxin assay kit (Lonza, Walkersville, MD, USA) and a HMGB1 ELISA kit (IBL International, Hamburg, Germany) according to instructions provided by the manufacturers.

### Terminal ileum permeability assay

Gut permeability was assessed using a method described previously with modifications [13]. Briefly, after one hour resuscitation, a 1-cm segment of ileum proximal to the cecum with intact superior mesenteric vessels was dissected. The two ends of the isolated ileum segment were ligated with 2–0 silk sutures. 0.2-ml of 0.1 M phosphate-buffered saline (PBS at PH 7.2) containing 25 mg/ml of FITC-dextran (FD-4; molecular weight 4000; Sigma-Aldrich) was injected into the lumen and blood samples were obtained 30 min after the FD-4 injection. Circulating FD-4 levels were determined with a Victor-3 multilabel counter (PerkinElmer Life and Analytical Sciences, Wallac Oy, Turku, Finland) at the excitation and emission wavelengths of 480 nm and 520 nm, respectively. Standard curves for calculating the FD-4 concentration in the samples was obtained by diluting various amounts of FD-4 in PBS.

### Lung Myeloperoxidase (MPO) activity

The MPO activity in the lung tissue was measured as an index of lung inflammation as previously described. Briefly, lung tissue was homogenized and sonicated in detergent buffer. The prepared samples were used in reactions for MPO activity determined spectrophotometrically (650 nm) by measuring hydrogen peroxide-dependent oxidation of 3, 3′,5,5′-tetramethylbenzidine [14].

### Pulmonary protein leakage

Evans blue dye (EB) was used to assess pulmonary protein leakage as previously described [15]. Briefly, mice were sacrificed with intravenous sodium pentobarbital (100 mg/kg) and blood was aspirated via cardiac puncture into a heparinized syringe for isolation of plasma 30 min after injected with Evan’s blue (EB, 0.4%). The pulmonary circulation was flushed with cold PBS. The lungs were excised, rinsed in PBS, blotted dry, snap frozen in liquid nitrogen, and stored at −80°C. The frozen tissue was homogenized in PBS (4°C) and incubated in formamide (60°C, 16 hours). After centrifugation (7,000 *g*) for 25 minutes at 4°C, the light absorption of the supernatant at 620 nm (A_620_) and 740 nm (A_740_) were recorded. Tissue EB content (μg EB/g lung tissue/minute) was calculated by correcting A_620_ for the presence of heme pigments: A_620_ (corrected) = A_620_ − (1.426 × A_740_ + 0.030) and comparing this value to a standard curve of EB in formamide/PBS.

### Generation of A-box

A-box was generated in our laboratory as previously described [8]. Briefly, plasmid (pGEX-5X-2) containing coding for A-box (a gift of Dr C-Y Wang, Medical College of Georgia, USA) or control plasmid was transformed into *E. coli* BL21 (DE3) and incubated in 2YT medium containing ampicillin (100 mg/mL) for 3–4 hrs at 37°C. Fusion protein A-box or GST was induced by 0.5 mM isopropyl D-thiogalctopyranoside and purified by using Glutathione Sepharose affinity column. The purified A-box or GST was passed over polymyxin B columns to remove any contaminating LPS.

### Statistics

Data are expressed as mean ± SEM. Statistical analysis was performed with two-way ANOVA followed by a Bonferroni correction for multiple comparisons. Graph Pad Software program was used for statistical analysis. A *p* value of less than 0.05 is considered to be statistically significant.

## Results

### HS/R induces intestinal barrier dysfunction

**Figure 1:**
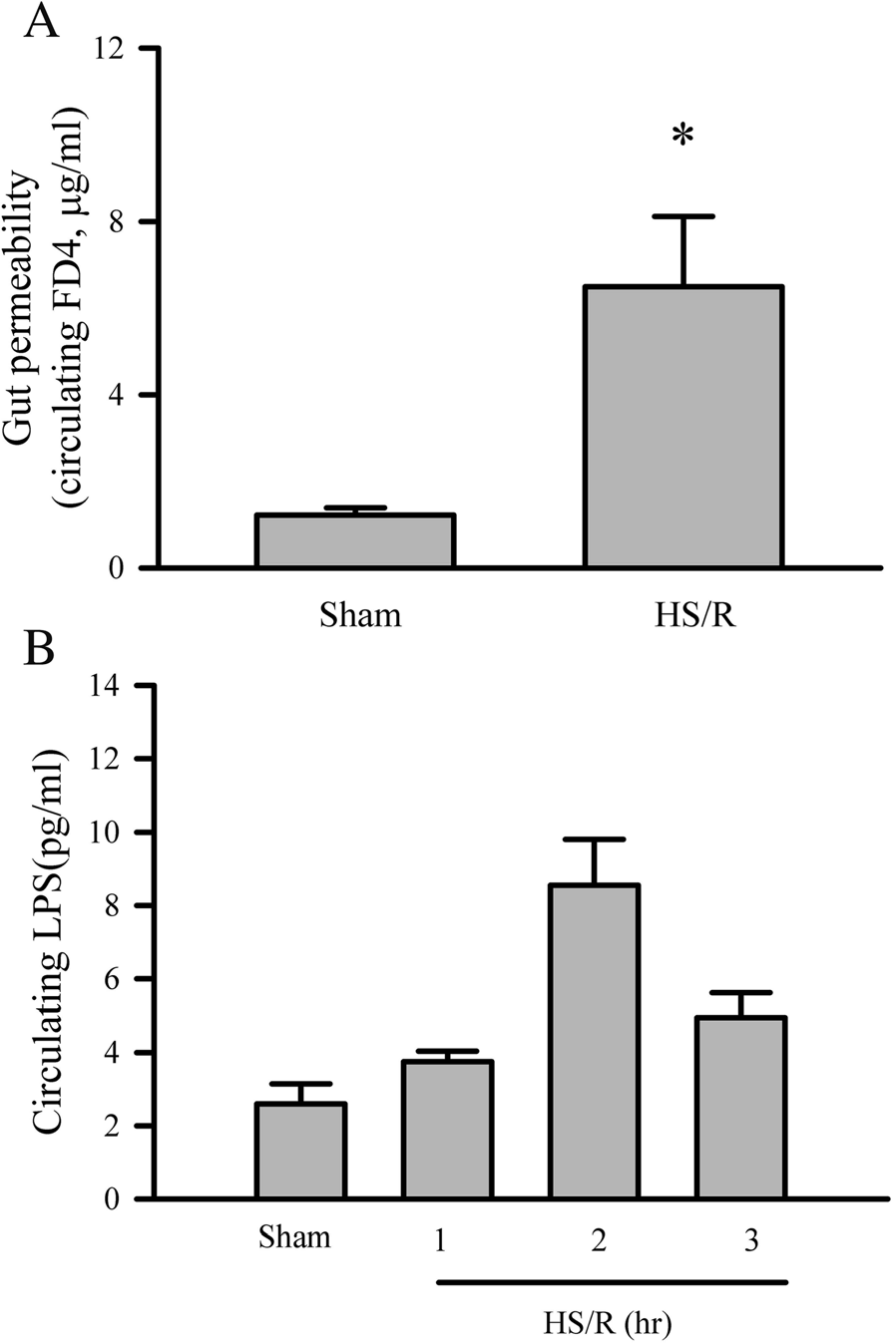
**Hemorrhagic shock and resuscitation results in increased mouse gut permeability and bacterial translocation into mouse circulation.** Mice were subjected to either sham procedure or hemorrhagic shock and resuscitation (HS/R). **A**. Gut permeability was assessed 30 min after completion of resuscitation. Five mice in each group, *p < 0.05 compared with sham group. **B**. Blood samples from sham or HS/R mice were collected at times indicated. Plasma levels of circulating LPS were determined with a Limulus Amebocyte Lysate (LAL) endotoxin assay kit. Six mice per group, *p < 0.05 compared with sham group.

Gastrointestinal system is the most vulnerable organ to be injured during the HS/R [16,17]. In order to determine whether HS/R results in intestinal barrier dysfunction manifested by an increase in gut permeability and bacterial leak into the circulation, we measured circulating levels of FD4 and LPS. As shown in Figure 1, circulating FD4 was increased 30 min post resuscitation, which was associated with increased circulating levels of LPS. The circulating LPS peaked at 2 hrs after the resuscitation.

### HS/R increases circulating HMGB1 and induces lung inflammation and pulmonary protein leakage

**Figure 2:**
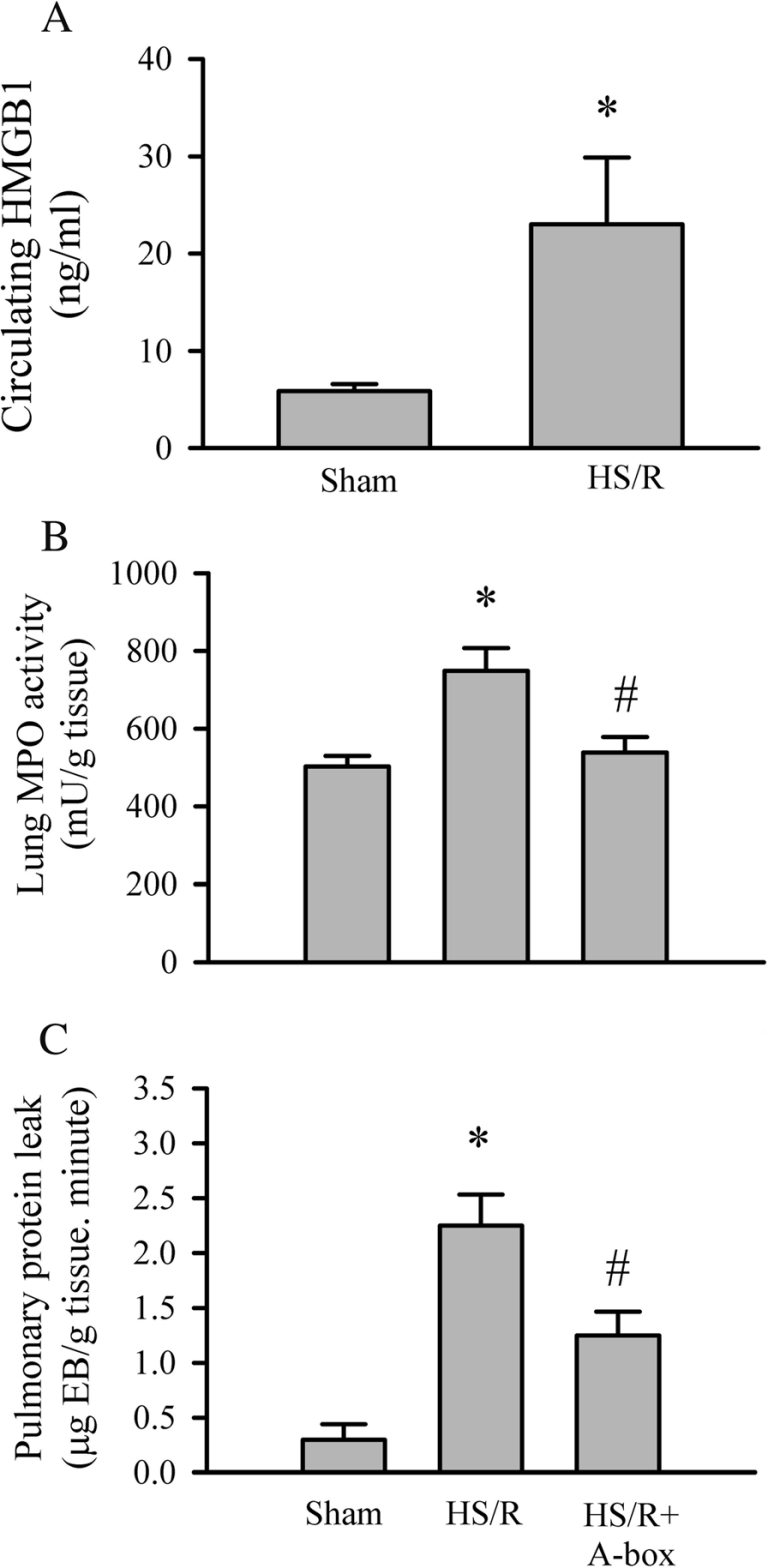
**HMGB1 mediated HS/R-induced acute lung injury (ALI).** Mice were subjected to sham surgery, HS/R, or HS/R plus A-Box (300 μg, i.p., given at the beginning of the transfusion of shed blood). **A**. Mouse blood was collected 24 hrs after resuscitation, plasma was obtained by centrifugation and circulating HMGB1 was determined with a HMGB1 ELISA kit. Five mice per group, *p < 0.05 vs sham group. **B** and **C**. Twenty-four hours after resuscitation, mouse ALI was assessed using tissue MPO activity **(B)** and pulmonary protein leakage **(C)**. Six mice per group for MPO activity experiments; Five mice in each group for pulmonary protein leakage experiments, *p < 0.0 *vs*. sham; ^#^p < 0.05 *vs.* HS/R.

HMGB1 is a cytokine involved in several inflammatory diseases [8,10]. Previous studies indicate endotoxemia results in its up-regulation of HMGB1 [8]. Thus, there is a possibility that intestinal dysfunction and bacterial translocation induced by HS/R can cause further organ dysfunction by induction of the HMGB1. As shown in Figure 2, circulating HMGB1 was increased 24 hrs after the HS/R (Figure 2A). Moreover, the HS/R challenge resulted in increased lung MPO activity and pulmonary protein leakage (Figure 2B and C).

### HMGB1 mediates the HS/R-induced acute lung injury

**Figure 3:**
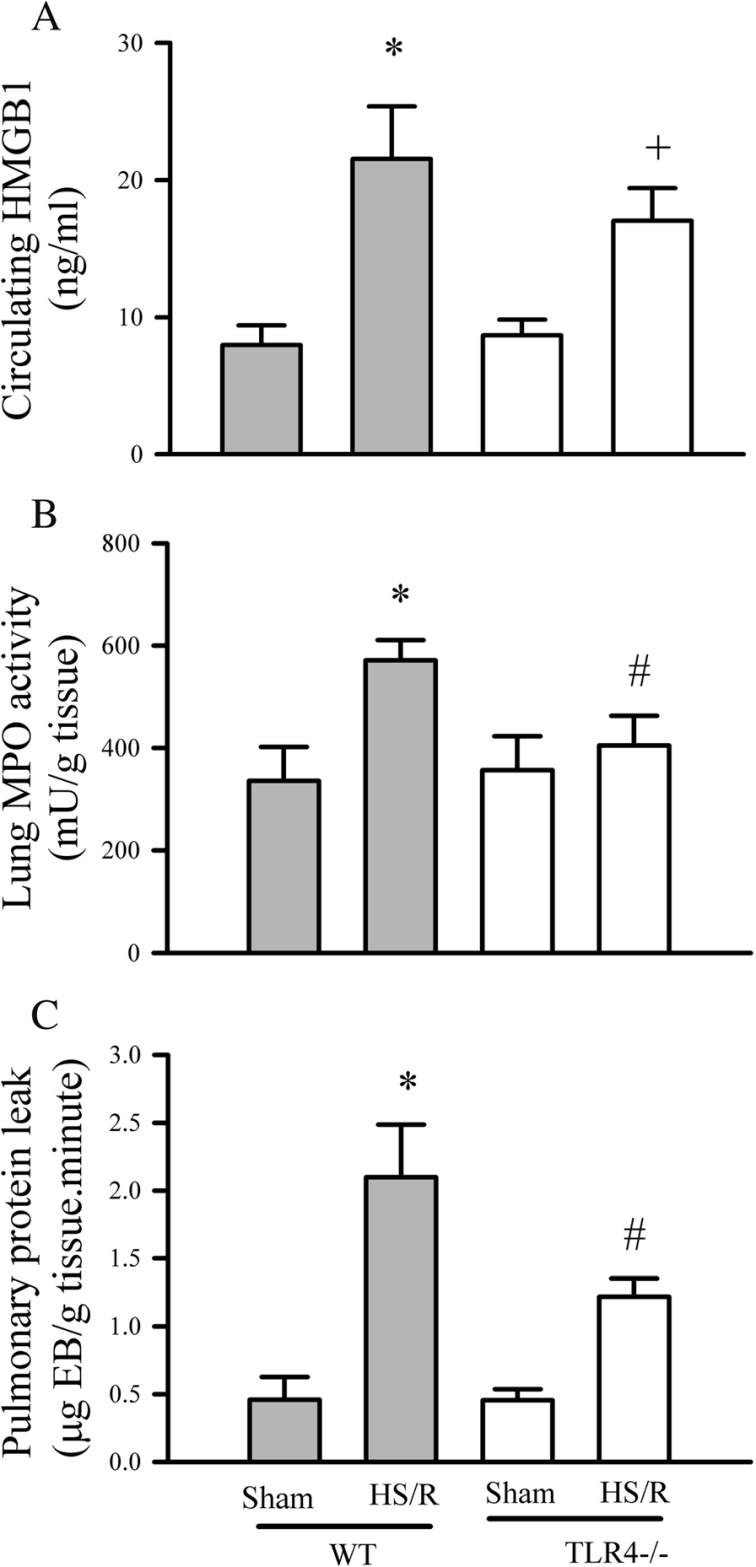
**Deficiency of TLR4 attenuates HS/R-induced acute lung inflammation.** Wild type (WT) and TLR4^−/−^ mice were subjected to either sham or HS/R and mouse circulating HMGB1 **(A)**, lung MPO activity **(B)** and pulmonary protein leakage **(C)** were determined 24 hrs after resuscitation. **A**. Five mice in each group, *p < 0.05 *vs.* WT sham; +p < 0.05 *vs.* TLR4^−/−^ sham; no difference between WT HS/R and TLR4−/− HS/R; B and **C**. Five mice in each group. *p < 0.05 vs WT sham, ^#^p < 0.05 vs WT HS/R.

In order to determine whether the HMGB1 mediates the HS/R-induced lung inflammation, mice with hemorrhagic shock were given A-box (300 μg/mouse) at the beginning of resuscitation with the RL and the lung MPO activity and pulmonary protein leakage were assessed. In addition, the HMGB1 receptor, TLR4^−/−^ gene knockout mice were challenged with sham or HS/R procedures, the circulating HMGB1 levels as well as lung MPO activity and pulmonary protein leakage were evaluated. As shown in Figure 2B and C, A-box treatment attenuated the HS/R-induced increase in lung MPO activity and prevented the pulmonary protein leakage. Moreover, as shown in Figure 3A, similar to the wild type counterparts, HS/R resulted in an increase in circulating HMGB1 in TLR4^−/−^ mice; however, the increase in lung MPO activity and pulmonary protein leakage were prevented in TLR4^−/−^ mice with HS/R as compared with the wild type controls.

### HMGB1 on gut injury in the early phase of HS/R

**Figure 4:**
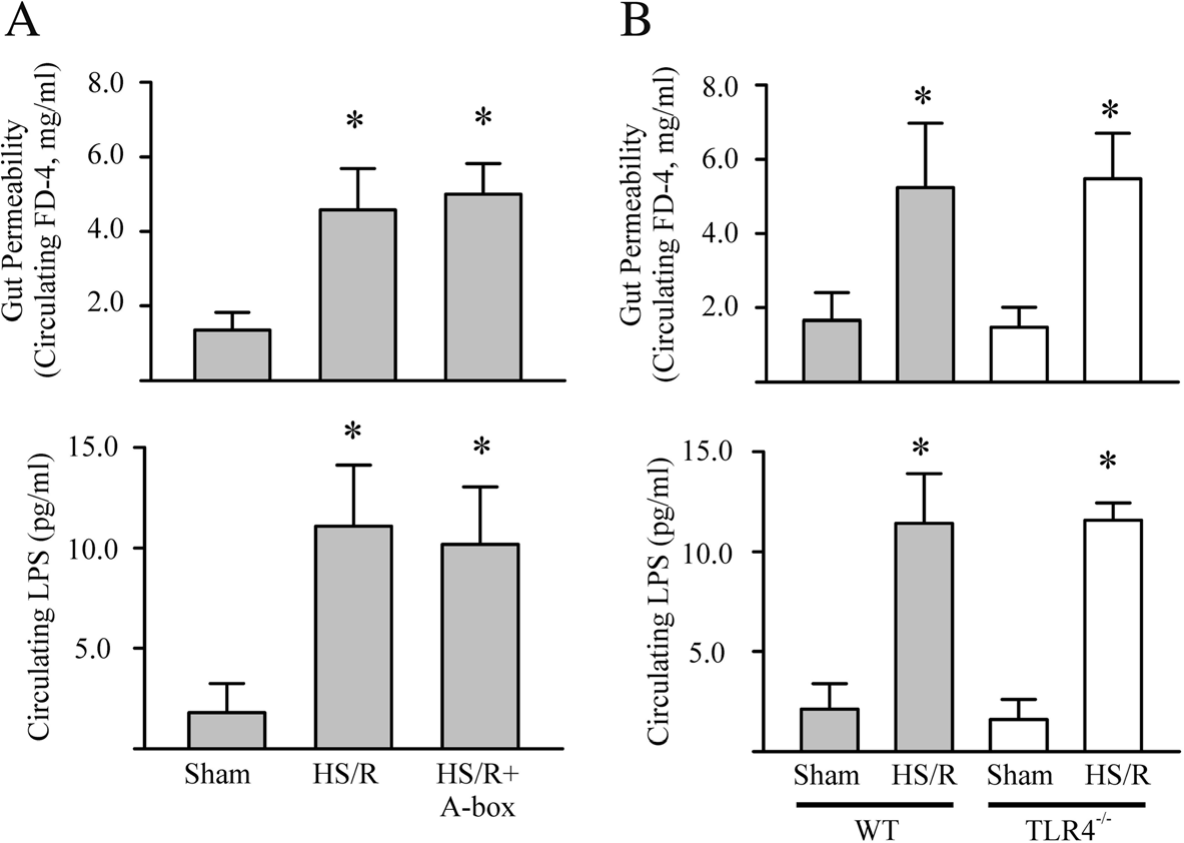
**Inhibition of HMGB1 does not show protection to gut injury in the early phase of HS/R in mice. A**. Mice were subjected to sham surgery, HS/R, or HS/R plus A-Box (300 μg, i.p.) given at the beginning of the transfusion of shed blood). Gut permeability and bacteria translocation were determined at 30 min and 2 hrs after completion of resuscitation by measuring circulating FD4 or circulating LPS, respectively. Five mice in each group, *p < 0.05 vs sham. **B**. Wild type (WT) and TLR4^−/−^ mice were subjected to either sham or HS/R. Gut permeability and bacteria translocation were determined at 30 min and 2 hrs after completion of resuscitation by measuring circulating FD4 or circulating LPS, respectively. Five mice in each group, *p < 0.05 vs related sham group.

To determine whether HMGB1 plays a role in the HS/R-induced gut injury/dysfunction, A-box was given to shock mice during the resuscitation stage and circulating levels of FD4 and LPS were determined at 30 min and 2 hrs after the resuscitation, respectively. In addition, mice deficient in TLR4 were challenged with HS/R, the circulating levels of FD4 and LPS were evaluated. As shown in Figure 4, pharmacologically inhibition of HMGB1 (A-box) (Figure 4A) or genetically inhibition of HMGB1 (TLR4 gene deletion) (Figure 4B) did not prevent the HS/R-induced gut injury/dysfunction.

## Discussion

In the present study, we demonstrate that the gut injury occurred after the resuscitation in a mouse model of HS/R as indicated by increased gut permeability (circulating FD-4 elevated). This result confirms our previous finding using a rat model of HS/R [12]. We further demonstrate that the gut injury in mice with HS/R leads to bacterial translocation into circulating system as evident by increased circulating levels of LPS. This data are also consistent with our previous work which demonstrates bacterial translocation to mesenteric lymph nodes due to gut injury [12]. A major function of the gut is to maintain a physical barrier to prevent absorption of toxin, antigen, and microorganisms. However, the gut is highly vulnerable to ischemic insult during the HS/R. We found increased gut permeability occurs as early as 30 min after resuscitation and causes bacterial translocation to circulating system which peaked at 2 hrs after the resuscitation.

A significant complication in patients following HS/R is acute lung injury (ALI) which is believed to be responsible for the high mortality of these patients [4]. Increased circulating levels of cytokines and induction of inflammation after resuscitation are considered to be the key steps in the development of acute ling injury [18]. Our data indicate that elevated serum LPS level due to intestinal dysfunction and bacterial translocation, and induction of HMGB1 secondary to hemorrhagic shock are important steps in the initiation of acute lung inflammation; inhibition of HMGB1 attenuates the HS/R-induced lung inflammation and pulmonary protein leak.

The cellular sources of increased HMGB1 in mice with HS/R are not quite clear. As the HMGB1 can be actively secreted by inflammatory cells and stressed parenchyma cells such as cardiomyocytes [8,11] hepatocytes and alveolar cells [19]. It can also be passively released by injured and necrotic cells [10]. One important cell type needs to note is natural killer T (NKT) cell within the liver. It has been reported that NKT quickly produces a wide array of cytokines under certain conditions to facilitate the liver as an important organ to modulate systemic inflammatory response [20]. Thus, we believe HMGB1 is produced by multiple sources in HS/R.

We have previously demonstrated that HMGB1 plays important roles in sepsis-induced myocardial dysfunction and mediates I/R-induced myocardial apoptosis [8,11]. HMGB1 has ability to engage with several receptors [10]. However, TLR4 is strictly required by HMGB1 for induction of cytokines expression and it is up regulated in the context of tissue injury in animal models [10]. Our results support this notion, HMGB1 involved in the HS/R-induced ALI by interaction TLR4, as inhibition of HMGB1 attenuated the ALI in the late phase of HS/R (Figure 2 and Figure 3). The exact mechanism by which the HMGB1 induces ALI remains not clear. It has been reported that HMGB1 is a chaperone protein which can greatly enhance the effect of IL-1β [21]. We have demonstrated that HMGB1 itself cannot induce cardiomyocyte apoptosis. However, HMGB1 potentiates the TNFα-induced cardiomyocyte apoptosis [11]. Therefore, we think HMGB1 involved in ALI through interaction with other cytokines.

The lung is an important organ targeted by inflammatory mediators following HS/R resulting in acute lung injury. Studies have confirmed that the pulmonary microvascular changes along with neutrophil infiltration and accumulation in the pulmonary vasculature in the early stages of HS/R [22,23]. The proposed “two-hit” hypothesis for acute lung injury revealed that HS/R primes the innate immune system for a second insult such as bacterial LPS to initiate an exaggerated inflammatory response in the lung [2]. Previous studies have demonstrated that HMGB1 can directly activate macrophages [24] and activated alveolar macrophages could produce chemokines to recruit PMN to the lungs and exaggerate lung inflammation and consequently ALI [25,26], but the exact mechanism(s) by which HMGB1 induces ALI remains unknown. Barness et. al. studied the relationship of TLR4 in mediating lung injuries following HS/R and hypothesized that TLR4 is needed for both the hemorrhagic shock and LPS-induced lung injury [27]. Thus, inhibition of HMGB1 function in HS/R might be an important step in prevention of acute lung injury inflammation/injury in the treatment of HS/R. The pathological sequence of events involved in HS/R are complex and not yet well-defined [4]. Consequently, ischemia/reperfusion and systemic inflammatory syndromes are two fundamental conventional events that results in systemic activation of immune system to the development of multiple organ dysfunctions.

## Conclusions

By using the mouse model of HS/R, we have demonstrated that gut permeability is increased during the hemorrhagic shock followed by resuscitation which causes bacterial translocation to circulating system. Increase in circulating bacterial LPS and tissue ischemia further results in increase in inflammation mediator HMGB1. HMGB1 appears to be an important mediator of acute lung injury that may be at least partly through interaction with TLR4. Thus, inhibition of HMGB1 may be a therapeutic target for the treatment of hemorrhagic shock/resuscitation-induced multiple organ dysfunctions.

## Abbreviations

EB: Evans blue dye
FD: FITC-dextran
NKT: Natural killer T cell
HS/R: Hemorrhagic shock and resuscitation
HMGB1: High mobility group box1 protein
LPS: Lipopolysaccharide
MAP: Mean arterial blood pressure
MOF: Multiple organ failure (MOF)
MPO: Myeloperoxidase
ROS: Reactive oxygen species
TLR: Toll-like receptor
RL: Ringer’s lactate
ALI: Acute lung injury
